# Dr. McNeil on Vaccination

**Published:** 1897-11

**Authors:** M. R. Leverson

**Affiliations:** Fort Hamilton, L. I., N. Y.


### DR. McNEIL ON VACCINATION.

FORT HAMILTON, L. I., N. Y., October 14th, 1897.

EDITOR OF THE HOMŒOPATHIC PHYSICIAN.

DEAR SIR:—Dr. A. McNeil's observations on vaccination in your issue of THE HOMŒOPATHIC PHYSICIAN for September serve to illustrate that chief defect in our schools which I have often pointed out and deplored, viz., that from the highest to the lowest they train the student in not thinking; in receiving upon "authority" instead of investigating for himself.

Even Hahnemann is not to be accepted blindly. Hahnemann, like most of his contemporaries, was deceived by the mendacious trick of the charlatan Jenner, whose tissue of previous falsehoods touching the cuckoo had not yet been unmasked. That series of falsehoods having procured him his F. R. S., he deliberately styled the disease known only as cow-pox, "Small-pox of the Cow," as though that were its usual name. This led Hahnemann as well as others to believe it was a similar disease, the truth being that it is really a syphilis or "syphilis of the cow."

The quotation from *The Organon* by Dr. McNeil is a sad blot upon that great work. It contains assumptions which are either erroneous, or at best not proven.

It is not true that either cow-pox or small-pox is auto-protective, or protective the one against the other. The small-pox eruption does not affect the lymphatics. The fever is not analogous, in small-pox it precedes the eruption, in cow-pox it follows it. Small-pox does not produce ophthalmia *per se*, while cow-pox and syphilis may. Where blindness follows small-pox it is from the neglect (sometimes unavoidable) of a pustule on the cornea.

I hope you will soon reach my note to Dr. Creighton's testimony before the Royal (British) Commission, and by publishing my pathological table help to clear up Dr. McNeil's difficulty.

Yours truly,

M. R. LEVERSON.

